# Legal aspects of privacy-enhancing technologies in genome-wide association studies and their impact on performance and feasibility

**DOI:** 10.1186/s13059-024-03296-6

**Published:** 2024-06-13

**Authors:** Alissa Brauneck, Louisa Schmalhorst, Stefan Weiss, Linda Baumbach, Uwe Völker, David Ellinghaus, Jan Baumbach, Gabriele Buchholtz

**Affiliations:** 1 Hamburg University Faculty of Law, University of Hamburg, Hamburg, Germany; 2 Interfaculty Institute of Genetics and Functional Genomics, Department of Functional Genomics, University Medicine Greifswald, Greifswald, Germany; 3 Department of Health Economics and Health Services Research, University Medical Center Hamburg-Eppendorf, Hamburg, Germany; 4 Institute of Clinical Molecular Biology (IKMB), Kiel University and University Medical Center Schleswig-Holstein, Kiel, Germany; 5 Institute for Computational Systems Biology, University of Hamburg, Hamburg, Germany

## Abstract

Genomic data holds huge potential for medical progress but requires strict safety measures due to its sensitive nature to comply with data protection laws. This conflict is especially pronounced in genome-wide association studies (GWAS) which rely on vast amounts of genomic data to improve medical diagnoses. To ensure both their benefits and sufficient data security, we propose a federated approach in combination with privacy-enhancing technologies utilising the findings from a systematic review on federated learning and legal regulations in general and applying these to GWAS.

**Supplementary Information:**

The online version contains supplementary material available at 10.1186/s13059-024-03296-6.

## Introduction

‘Privacy by design’ is an international principle of data protection law which stipulates that privacy measures must be built into the technical and organisational processes which handle personal data. This principle has been laid down in laws in different legislations, e.g. the European Union’s General Data Protection Regulation (GDPR) [1] or the California Consumer Privacy Act (CCPA) [2]. In particular, genomic data are highly sensitive [3]. For use in biomedical studies such as genome-wide association studies (GWAS), they often must be shared between institutions. Therefore, to achieve privacy compliance, researchers conducting such studies are required to implement privacy by design to achieve data self-determination. Essentially, this means that contractual agreements to respect privacy are not enough, but instead, researchers must reduce the possibility of privacy violations as much as possible, both through technology and appropriate organisational design. Privacy by design aims to institutionalise privacy at all levels, rather than tinkering with individual processes [4]. However, technology is developing rapidly and privacy by design principles, once formulated, are not necessarily sufficient to guarantee a satisfactory level of data protection in the long-term [5]. It is therefore not surprising that in practice, compliance with privacy by design, although necessary, is often perceived as a burden [6]. Challenges associated with the processing of genomic data—e.g. how privacy and research on genomic data can be harmonised, how genome-phenome investigations such as GWAS can be conducted without violating the privacy of the people involved and how individual or combined privacy-enhancing technologies (PET) can be used to meet privacy requirements—have repeatedly been the subject of many papers. For example, Berger and Cho [7] described the shift from traditional privacy approaches for sharing genomic data to advanced privacy-enhancing approaches and their challenges under data protection laws. Erlich and Narayanan [8] examined privacy breaches that are relevant to genomic information, e.g. attribute disclosure attacks via DNA (ADAD), which are particularly relevant for GWAS, as they are especially vulnerable to this form of attack, and appropriate risk mitigation strategies; these, however, do not refer to the legal requirement for privacy protection [8]. In their review, Bonomi et al. [9] analysed the privacy challenges associated with emerging applications for genetic testing performed directly by consumers and what techniques can protect privacy in the context of such analyses. Wan et al. [5] studied the regulations in the EU and the USA on the handling of genetic and genomic data and how the legal differences affect the use of such data, but do not provide a concrete analysis of the legal requirements. Shabani and Marelli [10] referred to codes of conduct or professional society guidance, i.e. ‘soft law’, in order to minimise the risks and offer the greatest possible legal protection for the handling of sensitive data such as genomic data and help to meet the requirements of the GDPR. Mitchell et al. [11] also discussed codes of conduct and additional certification mechanisms under Article 42 GDPR, giving a detailed overview of the legal framework under the GDPR and pointing out various difficulties, such as cross-border data transfers, how to deal with data relating to multiple genetic relatives or the right to rectification when genomic data is inaccurate. Other authors focus on the legal perspective: Quinn and Quinn [12] provided a general evaluation of genetic data under the GDPR and in regard to privacy by design, whilst Brauneck et al. [13] assessed federated learning and privacy-enhancing technologies (PETs) as measures to achieve GDPR compliance.

Our article diverges from prior work in that we trace the principle of privacy by design back to its legal basis and identify the requirements that need to be met before applying them specifically to GWAS on diseases and human traits. On this basis, we analyse each step of these studies and discuss the risks for data subjects associated with them as well as the legal downsides and merits of technical solutions before providing concrete advice on how to fulfil the privacy by design requirements of the GDPR. These requirements are enshrined in Article 25 GDPR and designed to safeguard data subjects’ rights, especially the right to informational self-determination. We focus on GWAS, however, the privacy by design concept applies to all types of studies in which genomic data from individuals are exchanged between different research sites for analysis purposes. We consider the same general privacy risk model as Wang et al. [14]. There are several known types of privacy attacks that are relevant to genomic data sharing, such as membership inference attacks [15, 16], attribution inference attacks [17] and reconstruction attacks [18]. Most commonly, attackers have access to the full or partial genomic sequences of the target and exploit side information, which usually increases the malicious potential of the attack significantly [14]. Our focus, however, is on general privacy risks, without focussing on specific attacks and aims to mitigate the privacy risks associated with the exchange of highly sensitive data through the use of privacy-enhancing techniques. First, we address the international and European background of privacy by design requirements, then demonstrate which challenges arise in research with genomic data, especially in GWAS with regard to GDPR requirements, and finally present recommendations for future GWAS in the form of privacy-enhancing technologies.

### Privacy by design and its impact on genome-wide association studies: a primer

GWAS aim to determine the impact of variation in the genome sequence on physical traits by identifying relationships between genetic variants and phenotypes, such as diseases, disease severity or other human traits. As a result, GWAS can both identify genetic risk factors and improve the standard of medical care [19]. The power of GWAS—especially when analysing common diseases and common variants (and with increasing sample sizes also rare diseases and/or low-frequency variants) —can most effectively be harnessed by studying large datasets from multiple centres with a very high number of participants. This requires data sharing amongst internationally distributed consortia [5, 20–24], which poses a number of legal challenges, not all of which are necessarily unique to GWAS, but result from the large number of participants and consequently large amounts of data that are required for performing GWAS. All of these challenges, which we will investigate in the following, can ultimately be traced back to the requirements of privacy by design.

Privacy by design is far from new [4]. There are many international examples of legislation on how privacy by design might be implemented. In the USA, this principle has been enshrined in, amongst others, the CCPA, and in 2012, the U.S. Federal Trade Commission (FTC), a regulator for antitrust and unfair trade practices, published a framework of privacy best practices for implementing privacy and data security for companies that collect and use consumer data [25]. This framework specifies ‘unfair’ and ‘deceptive’ practices as described by Sect. 5 of the FTC Act. The Commission takes action against companies ‘that promised consumers a certain level of security (in their privacy policies, for example) and then failed to deliver’ [4]. Another example of data protection laws is Japan’s Act on the Protection of Personal Information (APPI) [26], which was fundamentally revised in both 2017 and 2022 [27]. The APPI is partially similar to relevant EU laws, especially regarding the implementation of adequate security measures, in order to ease data transfers between Japan and the EU. Overall, it has a slightly narrower scope [27].

In Europe, privacy by design is explicitly required by the GDPR, the landmark regulation governing privacy protection and data use. The scope of what is meant by ‘privacy’ in the GDPR’s ‘privacy by design’ is different from the colloquial use. The GDPR lays out a number of ‘core principles’ beyond privacy (Article 5 GDPR), in the protection of which lies its raison d’etre. The principles with a particularly high relevance for GWAS are data protection and security, data self-determination and data fairness. The method of privacy by design (anchored in Article 25 GDPR) to protect the aforementioned principles is an obligation for systems that process personal data, which in turn is defined in Article 4 (1) GDPR as ‘any information relating to an identified or identifiable natural person (“data subject”)’. Genomic data therefore always constitutes personal data, since it is unique to each person (and thereby identifying) even if all other identifying information (e.g. name or address) is removed [10]. In practice, pseudonymised genomic data—and subsequently the study results concerning this data subject—can generally only be matched to a person whose genomic data are both accessible and linked to them, unless re-identification through relatives’ records in online genealogy services is possible—e.g. because they entered it into a database for ancestry services. This fact lowers the identification risks associated with genomic data. But the researchers cannot simply trust that the genomic data will not be linked to a natural person either. In light of this, the rapid rise of companies and business models that sell genetic data (e.g. for forensic analyses) directly to consumers raises new questions about data protection and ethics [5, 9, 28, 29]. Privacy can never be fully ensured and the consequences can as of yet not be fully anticipated. How real these risks of leaking genetic data are is shown, for example, by last year’s successful hacking attack that exposed 6.9 million users of the ancestry service 23 and Me [30], which resulted in a class action lawsuit against 23andMe for negligence and violation of the Illinois and California law [31]. The class lawsuit is based on allegations that the company failed to take reasonable security measures to protect its customers' sensitive data. If the class action is successful, the damage could amount to between 1 and 2 million dollars [32]. Similarly, violations of the GDPR may result in high fines or damages claims (Article 82 GDPR).

### Current practices and their legal issues

**Fig. 1:**
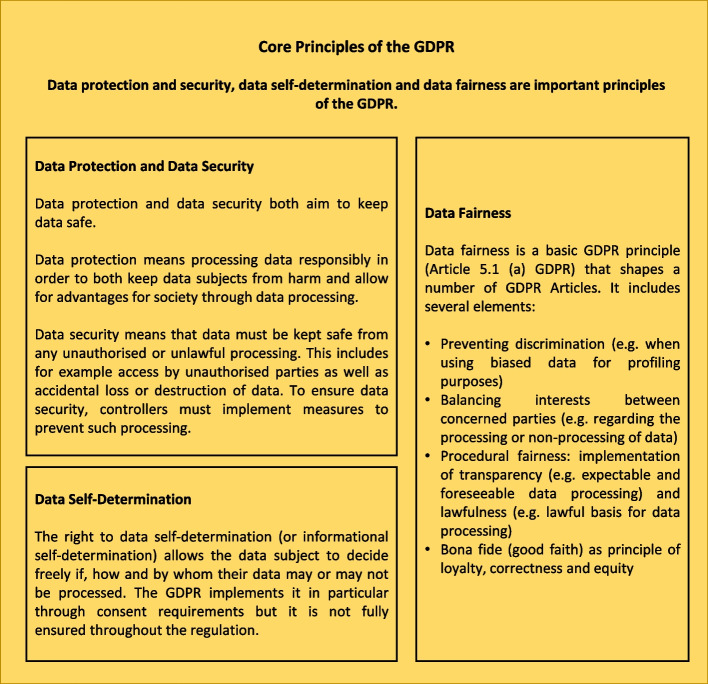
Core principles of the GDPR: overview over the GDPR principles of data protection and security [33] data self-determination [34] and data fairness [35] that have to be fulfilled by GWAS researchers

In our assessment of the compatibility of current GWAS practices with privacy by design requirements, we examine a number of legal issues that need to be addressed for the various data processing steps of a typical GWAS analysis. Especially relevant to GWAS practitioners are the legal challenges arising from the core principles of the GDPR: namely data protection and security, data self-determination and data fairness, all of which must be ensured through privacy by design. Figure 1 provides a general overview of the principles. Subsequently, we address specific challenges in a GWAS context.

Firstly, the exchange of genetic data is risky from a data protection and data security perspective, as individuals are identifiable by their genetic data (genetic fingerprint). This comes with a number of challenges that are (also) relevant in a GWAS context, of which we will explain four in more detail here:

1. Technical and organisational measures

Researchers, who are usually the party controlling the data (according to the GDPR: the ‘controller’ see Article 4 (7), Article 24 GDPR), must take ‘appropriate technical and organisational measures’ (Article 25.1 GDPR) to ensure data privacy and protection and minimise the risk of data breaches (i.e. accidental or unlawful destruction, loss or unauthorised disclosure of personal data) [13]. Due to the sensitivity of genomic data, data security should be embedded as an operating principle in the organisation (akin to a ‘safety first’ culture), and technical measures such as encryption and authentication/authorisation must be robustly implemented.

Some variants of GWAS approaches already include safety measures such as homomorphic encryption (HE) in their initial set-ups [36, 37]. And with regard to authentication/authorisation, trusted research environments (TREs) are an often-used option to prevent unauthorised access to de-identified data and/or re-identification of individuals [38]. A difficulty here that leads to legal challenges is that many research institutions and data providers use their own TREs for analysis purposes, so the data are often kept separately: Even if researchers have permission to use data from two separate TREs via multi-party TREs, it is often challenging to combine the data sets [39]. The reason for this are data use agreements that have to be negotiated. Measured by the size of the data set, the sensitivity of the data and the number of people who should have access to the data, these agreements are complex, time-consuming and therefore expensive.

Additionally, the necessary security standard is kept vague by both legislation and courts and has to be determined on a case-by-case basis which makes it difficult for practitioners to establish and adopt adequate security standards.

2. Security duration

Another data protection and security challenge is that personal data must be kept secure either until its deleted or for at least the duration of the data subject’s life [40], if not for that of close family members. The latter could be the case for genomic data: they differ from other personal data as they are directly linked to more than one person. No final decision on the status and rights of family members under the GDPR has been reached so far, but some scholars make strong—if controversial—cases that the need for data security does not diminish with the data subject’s death as far as the data reveals information about their relatives [41, 42].

3. Cross-border transfers

Depending on the location where research is to be conducted, additional difficulties for appropriate data protection arise from cross-border legislation transfers. This is particularly relevant for GWAS that are conducted in the EU and rely on the use of genotype imputation servers located in the USA [43, 44]. Imputation is used in almost every meta- or single GWAS study to combine data from different research sites and from different array/sequencing experiments. In this step of a GWAS, the data are still identifiable (Fig. 3, Step 3), and locally performed imputation by data protection-friendly genotype imputation servers located in the EU [45] is not always feasible. Regarding GWAS conducted in the USA, cross-border transfers are necessary if the study relies on data from EU subjects.

Two adequacy decisions by the European Commission, the so-called Safe Harbor Agreement and the so-called EU-U.S. Privacy Shield, have so far failed to provide a sufficiently secure basis for data transfers to the USA and were both declared invalid by the Court of Justice of the European Union (CJEU) (2015 Schrems I judgement [46] and 2020 Schrems II judgement [47]). Since July 10, 2023, the third adequacy decision, the so-called EU-U.S. Data Protection Framework (DPF), has been in force, covering all data transfers between the EU and the USA. This new adequacy decision will allow the transfer of personal data from the EU to the USA without the need for additional safeguards such as standard contractual clauses. To apply, it requires recipients in the USA to ‘join the DPF by committing to the DPF principles and self-certifying with the U. S. Department of Commerce’ [48]. The majority of public sector entities in the USA, as well as banks, airlines and insurance companies, are exempt from certification and therefore do not fall under the framework [49]. Data transfers to non-DPF-certified recipients require other safeguards in accordance with Article 46 GDPR (e.g. standard contractual clauses) [48, 50]. It remains to be seen whether the new adequacy decision will once again be challenged before the CJEU. The first private action to have the data protection framework agreement annulled was dismissed by the General Court of the European Union at the beginning of October last year. To our knowledge, the relevant U.S. imputation servers are not yet DPF-certified. For this reason, GWAS researchers who want to utilise U.S. imputation servers do not benefit from the advantages, in particular the intended legal certainty, that arise from the DPF. International imputation currently remains a data processing procedure that is legally complicated and often time-consuming. In lieu of the DPF, Article 46 GDPR mandates that appropriate safeguards must be taken and the European Commission published new standard contractual clauses in June 2021, which are mandatory for new contracts from 27 September 2021 [51]. This option requires more effort and time and lacks the benefit of legal certainty as to what constitutes appropriate safeguards that the DPF offers.

Furthermore, cross-border transfers require researchers to consider two legislations. Even though the GDPR is currently one of the strictest privacy laws in effect, it naturally does not cover every data protection and security provision under other jurisdictions.

4. Imputation methods

**Fig. 2:**
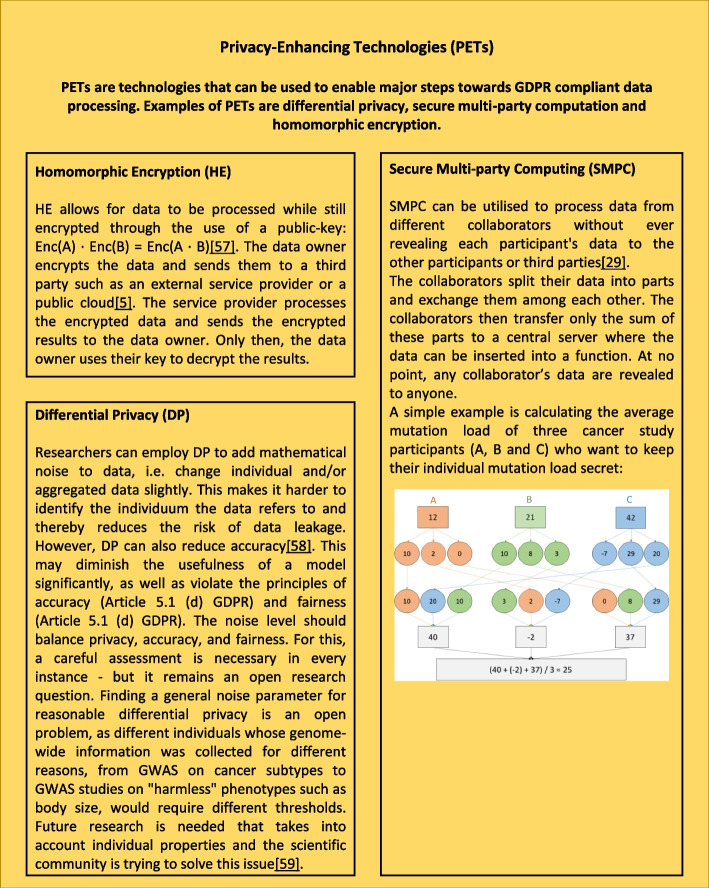
Overview of the function and aim of the three standard PETs: DP [60], SMPC [61] and HE [62] are three PETs that can—depending on circumstances alone or in combination—be used to fulfil privacy by design requirements. PETs can help to protect informational self-determination by ensuring that no unauthorised parties gain access to personal data. However, other data protection requirements, such as the principle of data fairness, are largely unaffected by PETs and must be ensured separately [63]

Imputation usually necessitates a data transfer to a third party. This leads to additional security risks. One way to guarantee such an adequate level of protection is provided by privacy-friendly genotype imputation methods. An example of such a privacy-friendly imputation method is *p-Impute*, which is based on HE [52]. *P-Impute* users can perform genotype imputation on encrypted genotype data and receive encrypted genotype outputs. A downside is that although the p-impute algorithm is faster due to the lack of a phasing step, it leads in its current form to lower accuracy for heterozygous SNPs [52]. Another HE-based method was presented by Kim et al. [53]. A comparison with state-of-the-art non-secure methods showed that HE-based solutions achieved comparable accuracy for common variants, but not for rare variants. An alternative to these HE-based frameworks are privacy-preserving imputation services based on trusted execution environment (TEE) technology, for example Intel SGX [54]. Due to the fact that it is hardware-based, the computational overhead is relatively small, as most of the computation is performed on the basis of the plaintext data inside the enclave, resulting in state-of-the-art imputation accuracy, which was significantly higher than HE-based solutions [54]. However, hardware-based solutions are not a homogenous concept in terms of trustworthiness [52, 55, 56] so they still often rely on users trusting the service provider to process sensitive data securely, which is not required with HE-based solutions [52]. They furthermore sacrifice some safety guarantees, which means that they do not have ‘the mathematically provable safety guarantees of HE' [55]. For further details on HE [5, 57] and other PETs [29, 58, 59], see Fig. 2.

Secondly, the participants’ data self-determination must be protected, especially in the form of consent. The GDPR creates several requirements for gaining consent for the processing of health and genetic data (Article 9.1 GDPR) and implementing measures to ensure the security of processing (Articles 24, 25, 32 GDPR). As a result, it is generally prohibited to process health and genetic data. The most prevalent exception to this rule is explicit consent (Article 9.2 lit. a GDPR). Consent management in GWAS though is becoming increasingly difficult due to the ever-growing number of participants in GWAS studies, with millions of participants already [64]. This becomes especially apparent in studies obtaining their data from biobanks. These may rely on very broadly worded consent forms to be effective and competitive, depending on the specific biobank collection—departmental collections, project-specific collections or hospital-wide collection [65, 66]. However, for compliance, consent must be very concrete and precise, explicitly permitting the use of genetic data and outlining the circumstances and possible future processing changes (as far as foreseeable in scientific terms). The concept of broad consent, that would allow for data to be processed in the context of yet unspecified projects and thereby allows for secondary use of data without a need for repeated consent, therefore generally conflicts with this need for specificity according to Article 5.1 lit. b GDPR [67]. Whilst recital 33 of the GDPR generally allows less strict requirements that would permit broad consent [67], the Article 29 Working Party (an independent advisory body to the European Commission) has signalled the prevalence of a stricter standard in 2017 (Working Party’s Guidelines on consent under Regulation 2016/679) [67]. However, broad consent is widely accepted by patients if it has been communicated as part of patient counselling [68]. Nevertheless, most patients do not wish to lose all control over their data and would prefer to make a new decision if research or processing circumstances change [66]. In any case, consent—including broad consent—needs a suitable information basis to be legitimate [69]. For this reason, patients should be informed as specifically as possible, in particular about whether ‘data is going to be shared with other research partners and across national borders’, whether linkage to registry data is to take place or whether research results or incidental findings are to be reported back [69]. Additionally, further complications arise from the fact that genomic data also always includes information about parents and close relatives, in particular in the case of monozygotic twins due to identical DNA, leading to yet unsolved issues regarding consent. At the moment, researchers can only achieve legal certainty if federal or state laws permit the processing of the data without consent [42] or the data processing is based on another processing basis in accordance with Article 9.2 GDPR.

Data can only be processed as long as valid consent exists. A later withdrawal of consent does not, however, affect the lawfulness of processing based on consent before its withdrawal (Article 7 (3) GDPR). Analyses carried out at the time of consent can thus continue to be used lawfully despite withdrawal. However, from the time of revocation, it is unlawful for parties to request the raw data for the purpose of verification or review. Consequently, researchers must take special legal and organisational measures to protect the participants’ (data) rights (see, for example: Politou et al. [70]).

In addition to consent, the self-determination right is also protected through transparency requirements (Articles 12–15 GDPR). An issue that may arise specifically in the context of GWAS and on which EU legislators have yet to make a decision is the right of a person to know or not to know about incidental genetic findings, i.e. cases in which scientists encounter genetic variants in their studies that affect a disease other than the one being studied [71]. Generally, this decision should be left to the data subject and is often also asked for when broad consent is obtained [68]. The question of how to deal with incidental findings is typically important when dealing with rare genetic variants (i.e. genetic variants with a low frequency in the population under study but high estimated risk of disease) or mutations in genes that are known to have a major impact on the development of a disease (e.g. genetic mutations in the breast cancer genes BRCA1 and BRCA2 usually have a major influence on the development of breast cancer). Information on incidental findings is therefore also particularly sensitive and must be protected.

Thirdly, genetic discrimination, violating the principle of data fairness, can occur when apparently population-specific risk factors are identified or when they incorrectly lead to systematically biassed (discriminatory) results for a particular population group (many GWAS studies listed in the GWAS Catalog of the National Human Genome Research Institute (NHGRI) [72] predominantly contains data on white populations) due to insufficient data precision for other ethnicities, for example in polygenic risk scores [73], which can, for example, lead to false prognoses [74, 75]. Furthermore, measures such as DP may skew the data leading to similar results or reinforcing bias [76]. To counteract this, the risks of DP have to be carefully considered before any amount of noise is added, and studies increasingly involve more specific populations from different parts of the world [74] or clearly point out the limitation of the study, namely that conclusions for other populations should be considered with caution [64].

### Comparison of the current genome-wide association study designs with regard to their privacy by design compatibility

**Fig. 3:**
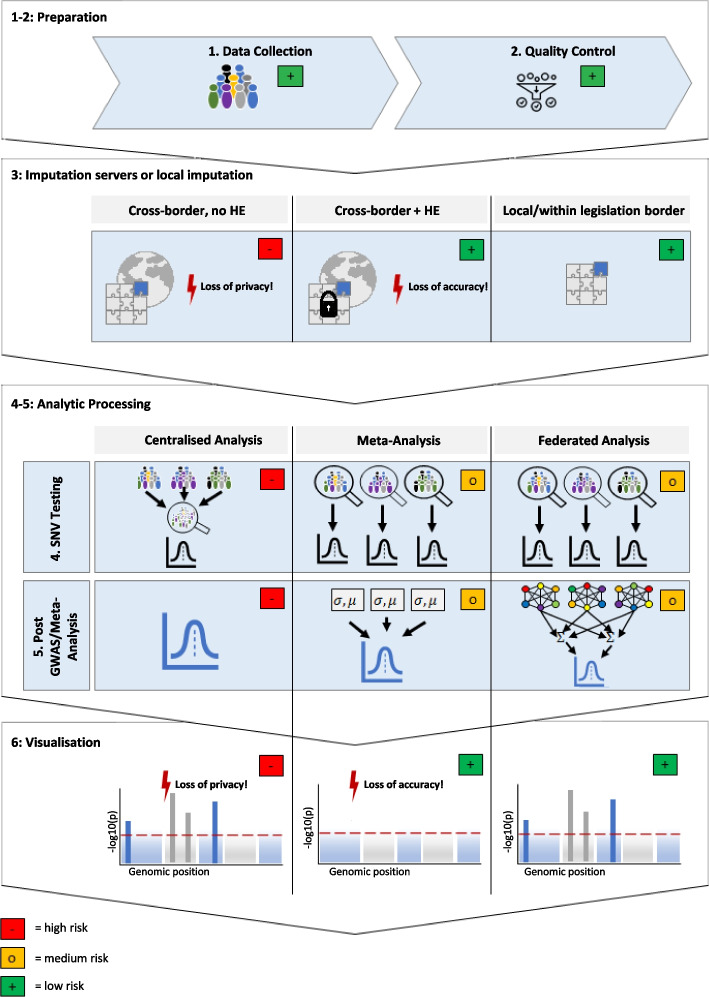
Graphical summary of typical GWAS data processing steps, inherent privacy risks and risks of reduced accuracy for centralised, meta and federated analysis. Data privacy during data collection (1) and quality control (2) can be ensured through the implementation of PETs, such as HE, DP and SMPC [77] and strict access control. There are no accuracy concerns. Data privacy standards during imputation (3) differ between international imputation servers and servers imputing locally or within a legislation border, such as EagleImp-Web (EU) [45], the Haplotype Reference Consortium (UK) [43] and TopMed [44]. Whereby local imputation can lead to a loss of accuracy in the case of obsolete references and algorithms, cross-legislation border transfers lead to additional privacy risks that can be combated through the use of HE, e.g. through the tool *p-Impute*. However, this still results in lower accuracy. Privacy risks and accuracy during the last three steps (4–6) depend on the GWAS study design: a centralised analysis is accurate but leads to privacy risks. A meta-analysis has a medium to low privacy risk but may suffer from reduced accuracy. A federated approach combines a medium to low privacy risk and high accuracy

In the following, we outline three different types of GWAS study designs and highlight certain special characteristics that need particular attention from a privacy by design perspective. The approaches differ in their data security and statistical power, especially in their robustness to data heterogeneity due to the heterogeneous nature inherent in biomedical data: (1) the centralised GWAS approach with all genotype data from different study populations pooled at one analysis site, (2) the meta-analysis approach, in which the genotypes from all participating studies are first analysed individually at participating centres and then only GWAS summary statistics are shared and meta-analysed and (3) the newly proposed federated analysis approach, in which genotype data stored separately at each institution is used to train local machine learning models which are then aggregated into one *federated* global model. Figure 3 provides a summary over the six typical GWAS data processing steps and inherent privacy and accuracy risks.

The responsibility to ensure privacy by design begins at the latest when researchers gain access to the genotype data (either at the quality control (step 2, see Fig. 3) or later) and continues for all processing steps where the data uniquely identify the data subjects. This is the case until the data are aggregated after single-nucleotide variant (SNV) testing and the retaining of any individual, privacy-sensitive information can be discarded (for meta-analysis and federated analysis approaches, this applies after step 4, see Fig. 3). The comparatively weaker privacy protection is a notable downside of central data processing (centralised analysis), which applies here up to step 6, see Fig. 3, if final figures or descriptive statistics are still produced using genotype data.

Since genomic data are inherently identifying, pseudonymisation alone is not sufficient to protect the rights of data subjects during these steps [11]. Therefore, researchers should additionally delete unnecessary and unusable data (e.g. local sample identifier) as soon as possible and deploy PETs such as DP, HE and SMPC to ensure secure communication and counter typical cyberattack schemes by design (see Fig. 2 for further details). It is crucial to evaluate and balance the trade-off between accuracy, computation time and data security due to the use of PETs carefully before choosing to apply—or forgo—any particular method [16, 58, 78]. If researchers utilise an already-established database (e.g. the UK Biobank [79]), the safe storage (as well as deletion after the end of the project) of the extracted data must be assured by the responsible third party, because individual study participants (e.g. from the UK Biobank) also regularly withdraw their consent.

Whilst aggregating individual data on centralised analysis servers is desirable from a research perspective, it massively increases data security risks (see Fig. 3, ‘loss of privacy’). Hence, separation of genotype data in distributed data silos is recommended from a legal point of view. Storing genomic data on large central servers also carries the risk of this data being stolen by hackers because in the event of a successful attack, a large amount of genomic data from a large number of individuals falls into the hands of the attacker all at once. There are two possible approaches suitable for conducting GWAS analyses on such distributed data: meta-analysis and federated GWAS. Meta-analysis aggregates the summary statistics for genomic loci for each of the distributed datasets, avoiding direct sharing of genomic data. However, this may come at the cost of accuracy for data sets with heterogeneously distributed class labels and confounding factors (e.g. uneven distribution of smokers across the data centres or unbalanced case–control ratios) [21, 80, 81] (see Fig. 3, Step 6). As mentioned at the beginning, this can lead to discriminatory outcomes which can, as well as exposing practitioners to legal risks, create real-world harm. Federated GWAS work through the emerging technology of federated learning, where statistical models are trained locally at each data centre, followed by a subsequent (or iterative) exchange of the model parameters either with a central server or with the other partners in a server-free manner, e.g. using SMPC, to produce a single joint model without exchanging the genotype data [21, 82]. Despite heterogeneously distributed phenotypes or confounding factors in different cohorts, in principle, the same results can be obtained as with centralised analysis, thus satisfying the requirements for accuracy [21] (see Fig. 3, step 6). An example for the accuracy of federated analysis was provided by Froelicher et al., whose Secure Federated Principal component analysis (SF-PCA) algorithm combines multiparty homomorphic encryption, interactive protocols and edge computing [83].

Whilst, as mentioned above, statistical scores such as the results of GWAS analyses generally do not have (directly) identifying qualities, there is a residual risk of revealing information about individual subjects; both the summary statistics in meta-analysis and the exchanged model parameters of federated GWAS may therefore in theory constitute personal data—in which case they would also require very stringent protection (although to a much lesser extent than the genomic data in centralised analysis). There is an ongoing debate in the scientific community if and to which degree one could re-identify an individual from a meta-GWAS’ summary statistics if, e.g. one knows a few hundred SNPs from that individual [16]. A simulated statistical attack showed that the presence of an individual in a GWAS cohort could be determined on the basis of the aggregated allele frequencies, provided the attacker has access to some raw genomic information about the individual in question [10, 15, 16, 84, 85]. The risk grows with the increasing collection of and access to (sometimes public) personal genetic marker data [85]. Another study demonstrated that genetic data can be matched with photographs, a risk that can be addressed by using DP for images [86]. According to the study, without access to high-quality, preferably three-dimensional images, the risk is small but not negligible, especially given the ever-developing camera and artificial intelligence (AI) technologies [86].

**Fig. 4:**
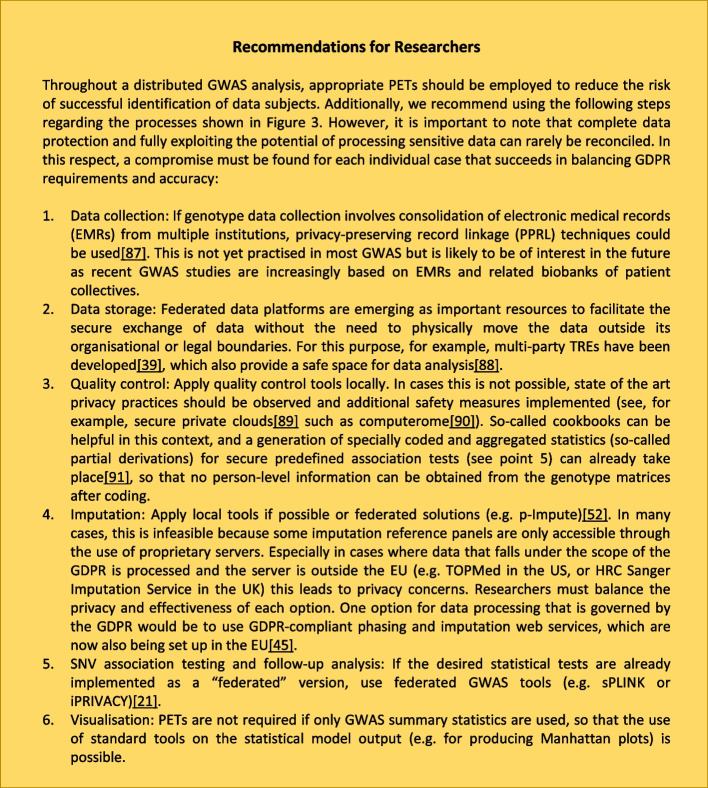
Overview of recommendations for researchers in relation to data collection, data storage, quality control, genotype imputation, SNV association testing and follow-up analysis and visualisation in distributed GWAS analysis

These findings make it even more important to implement safeguards to keep genomic data secure, especially when third parties (albeit exclusively for the intermediate storage of genomic data) are given access to provide services such as genotype imputation. Effective measures to mitigate such an attack on the trained statistical model itself include employing additional PETs that either add noise to the data (DP), specifically obfuscate the underlying data (by altering carefully selected linkage disequilibrium data) [16], generalise the data (by replacing values with general but semantically consistent ones) that suppress identifying data by removing specific values or by detecting and removing outliers before model training. These measures must be carefully chosen and administered to balance the accuracy-privacy trade-off in a way that is suitable for the sample populations’ specific features, such as demographics or outliers [59, 78] (see Fig. 4 for further details). Researchers must carry out this complex balancing process (e.g. have to choose the appropriate level of noise); in doing so, they are not bound by any specific legal requirements. Rather, they must protect the data in a way that corresponds especially to the state of the art, the probability of occurrence and the severity of the risks associated with the processing for the rights and freedoms of natural persons (Article 25.1 GDPR). At the same time, they must take into account technical developments that lead to both more secure measures and new risks, if they can be considered at all based on the characteristics of the study, to achieve the intended effect. In some cases, it might also be required to combine PETs to heighten their effectiveness and compensate for weaknesses. This especially pertains to DP as the amount of noise necessary to achieve adequate data privacy in the context of a GWAS is not reasonable with respect to accuracy concerns. This can be circumvented by combining DP with HE [5] or SMPC [36]. HE and SMPC profit in return from reduced communication and computation overhead [36]. Figure 4 shows which concrete actions can be taken when projects fall within the scope of the GDPR in the following six areas: data collection [87], data storage [39, 88], quality control [89–91], genotype imputation [45, 52], SNV association tests and follow-up analysis [21] as well as visualisation in distributed GWAS analysis.

## Conclusion

Fully ensuring privacy by design in GWAS comes with a number of challenges, but researchers who are conscious of these challenges and wish to tackle them head-on have access to a growing array of methods and tools. In particular, federated GWAS has the potential to overcome the most persistent privacy challenges for GWAS on distributed datasets and multi-centred GWAS meta-analysis whilst avoiding unacceptable accuracy trade-offs. However, it is hampered by legal uncertainties and a number of (yet) unresolved legal questions. The four most important ones which we discussed in our contribution are cross-border data transfers for genotype imputation purposes, the rights of family members regarding genomic data shared for study purposes, the legality of consent and a lack of diversity in studies of populations leading to genetic discrimination (or sometimes opens up discrimination, when genetic variation is studied only for certain population groups) [75]. Interestingly, contrary to previous assumptions, published GWAS summary statistics as stored in public databases such as the NHGRI GWAS Catalogue can reveal an individual’s participation in trait-specific GWAS. In the case of GWAS for diseases, this may lead to unwanted and unauthorised disclosure of information. For these and an array of further technical questions, guidelines and ultimately a robust legal framework are needed to provide legal certainty and improve compliance for the handling of genomic data in research under the GDPR regulatory regime. Data protection and privacy risks cannot be completely eliminated, but can be largely combated by implementing PETs and processing data locally where appropriate—these are fundamental aspects (along with self-determination and discrimination prevention) of privacy by design.

### Supplementary Information

Supplementary Material 1.
