# Author Correction: Legal aspects of privacy-enhancing technologies in genome-wide association studies and their impact on performance and feasibility

**DOI:** 10.1186/s13059-024-03311-w

**Published:** 2024-06-18

**Authors:** Alissa Brauneck, Louisa Schmalhorst, Stefan Weiss, Linda Baumbach, Uwe Völker, David Ellinghaus, Jan Baumbach, Gabriele Buchholtz

**Affiliations:** 1 Hamburg University Faculty of Law, University of Hamburg, Hamburg, Germany; 2 Interfaculty Institute of Genetics and Functional Genomics, Department of Functional Genomics, University Medicine Greifswald, Greifswald, Germany; 3 Department of Health Economics and Health Services Research, University Medical Center Hamburg-Eppendorf, Hamburg, Germany; 4 Institute of Clinical Molecular Biology (IKMB), Kiel University and University Medical Center Schleswig-Holstein, Kiel, Germany; 5 Institute for Computational Systems Biology, University of Hamburg, Hamburg, Germany

**Correction** **:** **Genome Biol 25, 154 (2024)**

**https://doi.org/10.1186/s13059-024-03296-6**

Following publication of the original article [1], the authors reported an error in the second equal contribution statement of their article. David Ellinghaus, Jan Baumbach and Gabriele Buchholtz are shared last authors. David Ellinghaus was erroneously omitted from this statement.

The original article [1] has been corrected.
