# Supplementary material for: Legal aspects of privacy-enhancing technologies in genome-wide association studies and their impact on performance and feasibility
Source: Genome Biol. 2024 Jun 13;25:154. doi: 10.1186/s13059-024-03296-6 (PMC11170858; doi:10.1186/s13059-024-03296-6)
Supplement: Supplementary file 1 — Supplementary Material 1. [file 13059_2024_3296_MOESM1_ESM.docx]

**Review history**

**First round of review**

**Reviewer 1**

This correspondence discusses the legal aspects of genome-wide association studies (GWAS) in the context of recent privacy regulations. The emphasis is on the core principles presented in GDPR, including: data protection and data security, data fairness, and data self-determination. Relevant issues that arise in typical GWAS study pipelines are presented. New methods based on privacy-enhancing technologies (PETs) are discussed as a possible solution to the legal issues. Recommendations for conducting GWAS with "privacy by design" are provided. The manuscript is clearly written overall. I have several suggestions and questions for clarification.

The contributions of this manuscript need to be better framed in the context of existing publications that also discuss the implications of GDPR for genomic data processing. Some example publications:
- https://doi.org/10.1038/s41431-017-0045-7
- https://doi.org/10.1038/s41576-022-00455-y
- https://doi.org/10.1007/s00439-018-1919-7

GWAS data are collected in different ways (e.g. biobanks vs. study-specific data collection). How do the legal implications differ among these settings?

The way GWAS is discussed in the manuscript seems to have a narrower scope than necessary for the purposes of this correspondence. E.g., imputation is common in GWAS, but is not a required step; whole-genome sequencing could also be used. The authors emphasize studies of common variants but analyzing rare variants is also common. It would be helpful to further distinguish GWAS-specific vs general conclusions about genomic data.

There are different types of "federated analysis" methods that are overlooked in the manuscript. For example the authors mention SMPC which has different security properties than a typical "federated learning" technique like secure aggregation. This reference proposes yet another approach based on HE: https://doi.org/10.1038/s41467-021-25972-y. These nuanced differences matter since they would affects how the methods align with the GDPR principles. For example, in Fig 1 classifying both meta and federated as medium risk may or may not be misleading depending on which technique is being referred to.

The comparison between federated GWAS and meta-analysis from a privacy standpoint has not been sufficiently discussed. How much risk is there in exchanging locally computed model parameters, and how does that compare to sharing summary statistics in meta-analysis?

There are other alternatives to p-impute: see https://doi.org/10.1016/j.cels.2021.07.010 and ahttps://doi.org/10.1016/j.cels.2021.08.001

The relevance of discussion about differential privacy for GWAS is unclear given that the required amount of noise is unlikely to be practical

Are there any implications of adopting PETs with respect to data fairness and data self-determination?

**Reviewer 2**

In this commentary/perspective, authors discuss different aspects of privacy by design (PBD) for the specific case of GWAS.

I like the focus on privacy-by-design principles since they are virtually never considered while developing methods other than efforts by some research groups. Therefore I believe this is a timely focus on an important message.

The manuscript is well written and I have several concerns and comments that i outline below. I hope these will help authors improve the manuscript.

1. Clear lack of many existing references and existing related works in the literature to many approaches that have addressed problems regarding GWAS, imputation, and federated methods that can be used to perform association studies.

2. Continuing previous comment, I am not able to put this perspective in context of many other reviews and perspectives on genetic privacy (google search returns 10s of these). How is the ideas here differ from more technical reviews that are outlined in previous reviews/commentaries?

3. Lack of threat models in GWAS; what types of practical attacks should create cause for concern? who would benefit an attack on a GWAS study? What motivates this attack? Are there any real cases that create cause for concern? What are the assumptions for these attacks to work?

4. Lack of focus on Community adoption: One of the main challenges is community adoption of PBD principles?

5. This statement seems fairly strong: "Genetic data always constitutes personal data, since it is unique to each person (and thereby identifying) even if all other identifying information (e.g. name or address) is removed11."

While I agree with the general PII aspect of genetic data, it is only identifying when an adversarial party can search for it somewhere, e.g., google, 23andme, ancestry etc. This requires a high level of expertise in genetic data before it really constitutes a privacy risk to reveal it. This is not as simple as searching for someone in yellow pages.

6. Lack of focus on Forensic usage: the usage of genetic data in forensic applications is rising at an alarming rate. There are clear ethical problems with this. Authors should discuss how PBD principles can help and/or limit forensic usage of genetic data in consumer and research domain.

7. Standardization of PBD principles (Table 1): I appreciate the effort to unify the privacy-by-design principles but I feel like they fall short and do not reflect challenges of GWAS studies.

8. "Current practices and their legal issues": This section discusses pooled, meta, and federated methods but does not further elaborate on their strength and shortcomings, i.e., lines 81 and 82 have an abrupt change in focus.

Also, there seems to be a lack of focus in this section although there is interesting points about GDPR's legal requirements about storage, transfers, and definition of consent. Specifically, authors discuss imputation (with lack of references) but then shift to US-EU data transfers. It may be useful to re-organize the section.

9. Lack of focus on family privacy: I think this statement is fairly vague : "Another data protection and security challenge is that data must be kept secure for at least the duration of the data subject's life17, if not for that of close family members."

The main issue here is which family members we should focus on? What are the ethical principles for family privacy?

10. I think Figure 1 is fairly vague in terms of risk and accuracy levels. It also oversimplifies a lot of the details. For example, I would argue following (I can be convinced otherwise): In majority of studies, meta-analysis provides very accurate results. I base this claim on the overwhelming number of studies that compared meta analysis with centralized analysis approaches. If you also look at the most recent GWAS studies, a large portion of them are meta-analysis-based studies.

**Reviewer 3**

Summary:
This article reviews the current legal standards for data protection, privacy, and security around the world and analyzes the extent to which current GWAS studies respect these standards.

The first section of the article introduces "privacy by design", a legal principle which stipulates that privacy protections must be included in the methods and technologies that organizations deploy. Because genetic data is by definition personally identifiable data, it comes within the purview of many "privacy by design" regulations including, for instance, the EU's GDPR law.

The article then summarizes current state-of-the-art approaches for federated GWAS analyses: (1) pooled and centralized GWAS analysis, (2) meta-analysis, and (3) federated analysis. The authors discuss the relative tradeoffs between these approaches from a privacy, accuracy, and efficiency perspective. The authors additionally discuss some risks associated with genetic data.

Strengths:
1. The authors have elucidated the core tradeoffs between the three approaches described well.
2. The legal principles they consider are broad, and include additional perspectives — such as fairness and discrimination concerns — which are, of course, related to privacy issues but not always discussed together with privacy/security concerns.
3. The authors additionally provide specific legal citations, especially within GDPR, to guide readers' understanding of each legal principle, such as, for instance, data self-determination.

Weaknesses:
1. Given the audience of Genome Biology, I believe this work could use more precise technical detail in the discussion of (1) privacy-enhancing technologies (Box 2), and (2) the precise legal consequences of these issues. I encourage the authors to draw upon recent legal battles, such as the 23andMe lawsuit, to inform readers concretely of potential genetic leaks and their consequences. Furthermore, the discussion of PETs in Box 2 could be more precise. For instance, it is unclear what "DP adds mathematical noise to data" means. What kind of data? Aggregated data or individual data? Who is adding the noise? Furthermore, is there any legal agreement on how to choose these noise levels?
2. More discussion about genetic data privacy and GWAS outside of the EU's GDPR would be appreciated. While the GDPR is the landmark law for genetic privacy, several other countries have passed laws and it'd be good to understand how the principles introduced in Box 1 apply more broadly in other legislation.

Comments:
This is a personal opinion, and I am happy to be convinced otherwise, but I think this article could use some more hedging. The consequences of genetic data breaches are unknown and unclear, and this should be acknowledged. Some leakage will always be likely, and it should be noted that there is significant uncertainty on the consequences of genetic privacy. Lines 217-225 are applicable to a broad range of data. Many data modalities contain personally identifiable and extractable information which could be linked to, for instance, photographs. Social media profiles, which people often publish publicly, could easily be linked to photographs or other modalities. I would like to see more discussion, or would be happy to engage in a discussion with the authors, on the uniqueness and necessity of GWAS privacy.

The following citations are omitted but are sufficiently related:
[1] Blatt, Gusev, Polyakov, and Goldwasser, PNAS 2020.
[2] Froelicher et al, Nature Communications 2021.
[3] Johnson and Shmatikov, ACM SIGKIDD 2013.
[4] Froelicher et al, IEEE S&P 2023.


Stylistic Questions:
The Genome Biology Correspondence guidelines mention that articles should contain no more than 15 references and no more than 2 figures. They also should relate directly to a work previously published in the journal. Of course it is up to the Editors, but this article does not seem to fit at a Correspondence.

**Authors’ response to reviewers**

We thank the editor and the reviewers for their comments and suggestions to improve our paper. We believe that they helped significantly improve the quality of the manuscript and look forward to further comments, if any. All the changes we have made in the paper have been highlighted in yellow.

Reviewer 1 - comment 1
The contributions of this manuscript need to be better framed in the context of existing publications that also discuss the implications of GDPR for genomic data processing. Some example publications:
- https://doi.org/10.1038/s41431-017-0045-7
- https://doi.org/10.1038/s41576-022-00455-y
- https://doi.org/10.1007/s00439-018-1919-7

Our response: Thank you very much for this suggestion. We agree, and now reference all three publications and added more context about the existing publications discussing the impact of the GDPR on GWAS and the processing of genomic data in general.

Reviewer 1 - comment 2
GWAS data are collected in different ways (e.g. biobanks vs. study-specific data collection). How do the legal implications differ among these settings?

Our response:
Thank you very much for pointing this out. The foremost difference is the reliance of many biobanks on broad consent. However, the general interest in this consent form, the legal uncertainties and data security requirements remain the same. Without sufficient data security, biobanks risk losing the trust of data subjects which is crucial to their continued existence. Recent events in connection with the UK Biobank show just how fragile this trust is. (https://www.ukbiobank.ac.uk/learn-more-about-uk-biobank/news/response-to-highly-misleading-article-in-the-observer). We added some information on the relevance of broad consent for biobanks and its implication for their competitiveness.

Reviewer 1- comment 3
The way GWAS is discussed in the manuscript seems to have a narrower scope than necessary for the purposes of this correspondence. E.g., imputation is common in GWAS, but is not a required step; whole-genome sequencing could also be used. The authors emphasize studies of common variants but analyzing rare variants is also common. It would be helpful to further distinguish GWAS-specific vs general conclusions about genomic data.

Our response:
We agree with the reviewer that in genetic epidemiology, genetic researchers test for association between human traits and diseases and genetic variation (most often SNP alleles) across the full spectrum of their frequency (common, low or rare) in the general population. We did not intend to differentiate between common and rare genetic variants but wanted to point out the different importance of rare and common variants in the context of incidental findings on page 9. Therefore, we have added the following sentence on page 9 and deleted the previous sentence with the distinction between rare and common variants:
"The question of how to deal with incidental findings is typically important when dealing with rare genetic variants (i.e. genetic variants with a low frequency in the population under study but high estimated risk of disease) or mutations in genes that are known to have a major impact on the development of a disease (e.g. genetic mutations in the breast cancer genes BRCA1 and BRCA2 usually have a major influence on the development of breast cancer). Information on incidental findings is therefore particularly sensitive and must be protected."

We also agree that, in general, any genetic study that deals with genome-wide or partially genome-wide data, whether generated by genotyping or sequencing technologies, must address the protection of genetic data. Because most scientists and potential readers of our articles are at least familiar with the study design of a GWAS study, which is one of the most commonly used study types in genetic epidemiology nowadays and in which data are commonly exchanged on a large scale between different research sites, the GWAS setting is a valuable example to emphasise the importance of protecting genetic data. We have included the following sentence in the introduction to make this more understandable:

"We focus on GWAS, however, the privacy by design concept applies to all types of studies in which genomic data from individuals are exchanged between different research sites for analysis purposes."
Because genotype phasing and imputation is performed in almost every meta- or single GWAS study to combine data from different research sites and from different array/sequencing experiments (e.g. recently also performed for data from whole-exome sequencing experiments) to allow investigations across datasets based on the same genetic marker content, and because this process is nowadays almost always carried out by computationally powerful imputation servers, on which the genetic data must be uploaded, it is important to consider the issue of genotype imputation in the context of legal aspects and security techniques for data protection. To make this clear we added the following sentence on page 6: “Imputation is used in almost every meta- or single GWAS study to combine data from different research sites and from different array/sequencing experiments.”

Reviewer 1 - comment 4
There are different types of "federated analysis" methods that are overlooked in the manuscript. For example the authors mention SMPC which has different security properties than a typical "federated learning" technique like secure aggregation. This reference proposes yet another approach based on HE: https://doi.org/10.1038/s41467-021-25972-y. These nuanced differences matter since they would affects how the methods align with the GDPR principles. For example, in Fig 1 classifying both meta and federated as medium risk may or may not be misleading depending on which technique is being referred to.

Our response:
Thank you very much for your comment. The field of "federated analysis" is highly relevant and is developing at an enormous speed. For this reason, there is a rapidly growing number of publications and approaches in the scientific community. Therefore, we chose to give an overview over the main methods and to discuss security measures such as HE separately as they can be combined with these methods. We now mention other approaches, like the suggested approach based on HE (https://doi.org/10.1038/s41467-021-25972-y) or an approach based on multiparty HE, interactive protocols, and edge computing (https://www.researchgate.net/publication/369759790_Scalable_and_Privacy-Preserving_Federated_Principal_Component_Analysis).

Reviewer 1 - comment 5
The comparison between federated GWAS and meta-analysis from a privacy standpoint has not been sufficiently discussed. How much risk is there in exchanging locally computed model parameters, and how does that compare to sharing summary statistics in meta-analysis?

Our response:
Thank you very much for your comment. Both the exchange of locally calculated model parameters and the exchange of summarized statistics in the meta-analysis can be classified as safe from a privacy point of view. In this respect, the two GWAS approaches do not differ. The situation is somewhat different with regard to accuracy. Here, meta-analyses in GWAS perform worse on heterogeneously distributed data. We address the accuracy problem in the paper.

Reviewer 1 - comment 6
There are other alternatives to p-impute: see https://doi.org/10.1016/j.cels.2021.07.010 and ahttps://doi.org/10.1016/j.cels.2021.08.001

Our response:
Excellent point. We have added the suggested sources.

Reviewer 1 - comment 7
The relevance of discussion about differential privacy for GWAS is unclear given that the required amount of noise is unlikely to be practical.

Our response:
We agree. It is indeed unclear under which circumstances differential privacy can be deployed effectively in a GWAS-setting. It’s an open question and the research community is currently still working on ways to combine differential privacy with GWAS (e.g. https://www.ncbi.nlm.nih.gov/pmc/articles/PMC4623434/). We now address this issue in the article.

Reviewer 1 - comment 8
Are there any implications of adopting PETs with respect to data fairness and data self-determination?

Our response:
Thank you very much for your comment. We’ve now included remarks on the connections between PETs and both data fairness and self-determination: differential privacy may influence data fairness negatively due to the potentially significant accuracy loss and PETs in general can have positive - if only indirect - impact on self-determination.

Reviewer 2 - comment 1
Clear lack of many existing references and existing related works in the literature to many approaches that have addressed problems regarding GWAS, imputation, and federated methods that can be used to perform association studies.

Our response:
Thank you for the comment. We agree that our work needed to make better reference to existing literature on this subject in order to demonstrate how our paper differs from it. Therefore, we’ve added the following sentences on page 2 to cover this aspect:
“Challenges associated with the processing of genomic data – e.g. how privacy and research on genomic data can be harmonised, how genome-phenome investigations such as GWAS can be conducted without violating the privacy of the people involved, and how individual or combined privacy-enhancing technologies (PET) can be used to meet privacy requirements - have repeatedly been the subject of many papers. For example, Berger and Cho[1] described the shift from traditional privacy approaches for sharing genomic data to advanced privacy-enhancing approaches and their challenges under data protection laws. Erlich and Narayanan[2] examined privacy breaches that are relevant to genomic information, e.g. attribute disclosure attacks via DNA (ADAD), which are particularly relevant for GWAS, as they are especially vulnerable to this form of attack, and appropriate risk mitigation strategies; these, however, do not refer to the legal requirement for privacy protection[2]. In their review, Bonomi et al.[3] analysed the privacy challenges associated with emerging applications for genetic testing performed directly by consumers and what techniques can protect privacy in the context of such analyses. Wan et al.[4]. studied the regulations in the EU and the United States on the handling of genetic and genomic data and how the legal differences affect the use of such data, but do not provide a concrete analysis of the legal requirements. Shabani and Marelli[5] referred to codes of conduct or professional society guidance, i.e. "soft law", in order to minimise the risks and offer the greatest possible legal protection for the handling of sensitive data such as genomic data and help to meet the requirements of the GDPR. Mitchell et al.[6] also discussed codes of conduct and additional certification mechanisms under Article 42 GDPR, giving a detailed overview of the legal framework under the GDPR and pointing out various difficulties, such as cross-border data transfers, how to deal with data relating to multiple genetic relatives or the right to rectification when genomic data is inaccurate. Other authors focus on the legal perspective: Quinn and Quinn[7] provided a general evaluation of genetic data under the GDPR and in regard to privacy by design, while Brauneck et al.[8] assessed federated learning and privacy-enhancing technologies (PETs) as measures to achieve GDPR compliance.
Our article diverges from prior work in that we trace the principle of privacy by design back to its legal basis and identify the requirements that need to be met before applying them specifically to GWAS on diseases and human traits. On this basis, we analyse each step of these studies and discuss the risks for data subjects associated with them as well as the legal downsides and merits of technical solutions before providing concrete advice on how to fulfil the privacy by design requirements of the GDPR.”

Reviewer 2 - comment 2
Continuing previous comment, I am not able to put this perspective in context of many other reviews and perspectives on genetic privacy (google search returns 10s of these). How is the ideas here differ from more technical reviews that are outlined in previous reviews/commentaries?

Our response:
Thank you for the comment. While more technical reviews focus on some of the same issues we do - data security, accuracy - they largely lack legal assessments, especially regarding the principles of privacy by design and its anchoring in data protection law. However, such assessments are necessary in order to get from a vague idea of data protection to concrete legal requirements for the processing of sensitive data to minimise the risk of liability. For this reason, we believe that we are making an important contribution to the scientific debate on GWAS and privacy by design principles with a focus on the GDPR. We now differentiate our paper from others on page 2 (see our answer to comment 1). In light of the new adequacy decision between the EU and the US, we have further expanded on legal considerations in the section “Current practices and their legal issues”, e.g. regarding data transfers between the EU and the US. In view of this recency of this topic, there are no publications on the current legal status and specific details on the practical implications.

Reviewer 2 - comment 3
Lack of threat models in GWAS; what types of practical attacks should create cause for concern? who would benefit an attack on a GWAS study? What motivates this attack? Are there any real cases that create cause for concern? What are the assumptions for these attacks to work?

Our response:
Thank you for your comment. We agree that our work needs to be more specific about the attacks and the risks involved. Therefore, we’ve added the following sentences on page 2 to cover this aspect:
“We consider the same general privacy risk model as Wang et al.[9]. There are several known types of privacy attacks that are relevant to genomic data sharing, such as membership inference attacks[10,11], attribution inference attacks[12] and reconstruction attacks[13]. Most commonly, attackers have access to the full or partial genomic sequences of the target and exploit side information, which usually increases the malicious potential of the attack significantly[9]. Our focus, however, is on general privacy risks, without focussing on specific attacks, and aims to mitigate the privacy risks associated with the exchange of highly sensitive data through the use of privacy-enhancing techniques.”

To our knowledge, there are no publicly known explicit attacks on GWAS data on a large scale, just as there are no explicit and systematic attacks on omics data. However, genetic data from GWAS studies are genome-wide data of over one million genetic markers and are therefore personal data for the unique identification of a person (genetic fingerprinting; special case of identical twins omitted). A possible loss of GWAS data is a serious data loss of personal data, see also the current case of 23andMe, see comment to reviewer #3, comment #1.

Reviewer 2 - comment 4
Lack of focus on Community adoption: One of the main challenges is community adoption of PBD principles?

Our response:
Thank you very much for pointing this out. The vague requirements for PBD are indeed a problem that has yet to be solved. To make this clearer, we’ve added the following sentences on pages 1-2 and 6 to cover this aspect:
“However, technology is developing rapidly and privacy by design principles, once formulated, are not necessarily sufficient to guarantee a satisfactory level of data protection in the long term[4]. It is therefore not surprising that in practice, compliance with privacy by design, although necessary, is often perceived as a burden[14], (...)”
“Additionally, the necessary security standard is kept vague by both legislation and courts and has to be determined on a case-by-case basis which makes it difficult for practitioners to establish and adopt adequate security standards.”
To enable community adoption in the long term, we believe that first legal uncertainties must be removed. To do this, the European legislator must take action or the jurisdiction on this matter provides clarity. And second clear and easy-to-follow guidelines are needed on how the PBD principles should/could be implemented. To this end, we provide an “Overview of recommendations for researchers in relation to data storage, data control, genotype imputation, SNV association testing and follow-up analysis and visualisation in distributed GWAS analysis.”

Reviewer 2 - comment 5
This statement seems fairly strong: "Genetic data always constitutes personal data, since it is unique to each person (and thereby identifying) even if all other identifying information (e.g. name or address) is removed11."
While I agree with the general PII aspect of genetic data, it is only identifying when an adversarial party can search for it somewhere, e.g., google, 23andme, ancestry etc. This requires a high level of expertise in genetic data before it really constitutes a privacy risk to reveal it. This is not as simple as searching for someone in yellow pages.

Our response:
We agree that we should include the practical considerations to put the legal assessment of genetic data as identifying into perspective. Therefore, we’ve added the following sentences on page 3 to cover this aspect:
“In practice, pseudonymised genomic data - and subsequently the study results concerning this data subject - can generally only be matched to a person whose genomic data are both accessible and linked to them - e.g. because they entered it into a database for ancestry services. This fact lowers the identification risks associated with genomic data. But the researchers cannot simply trust that the genomic data won’t be linked to a natural person either.”

Reviewer 2 - comment 6
Lack of focus on Forensic usage the usage of genetic data in forensic applications is rising at an alarming rate. There are clear ethical problems with this. Authors should discuss how PBD principles can help and/or limit forensic usage of genetic data in consumer and research domain.

Our response:
Thank you for the comment. We agree that there is a particular risk regarding the potential of forensic analyses that should be mentioned. Therefore, we’ve added the following sentence on page 3 to cover this aspect:
“In light of this, the rapid rise of companies and business models that sell genetic data (e.g. for forensic analyses) directly to consumers raises new questions about data protection and ethics[3,15].”

Reviewer 2 - comment 7
Standardization of PBD principles (Table 1): I appreciate the effort to unify the privacy-by-design principles but I feel like they fall short and do not reflect challenges of GWAS studies.

Our response:
Thank you for your comment. We now differentiate between a general overview of the PBD principles in the table and the challenges of GWAS studies within the text.

Reviewer 2 - comment 8
"Current practices and their legal issues": This section discusses pooled, meta, and federated methods but does not further elaborate on their strength and shortcomings, i.e., lines 81 and 82 have an abrupt change in focus.
Also, there seems to be a lack of focus in this section although there is interesting points about GDPR's legal requirements about storage, transfers, and definition of consent. Specifically, authors discuss imputation (with lack of references) but then shift to US-EU data transfers. It may be useful to re-organize the section.

Our response:
Thank you for your comment. In the section “Current practices and their legal issues” we now focus solely on the challenges GWAS generally face in regards to privacy by design. We moved the discussion regarding the three methods to the section “Comparison of the current genome-wide association study designs with regard to their privacy by design compatibility” where it is followed by an examination of their individual strengths and weaknesses.
With regard to imputation, we now provide detailed information on the legal challenges and have backed this up with references. We have added the following sentence on page 6:

“This is particularly relevant for GWAS that are conducted in the EU and rely on the use of genotype imputation servers located in the U.S. [43,44]. Imputation is used in almost every meta- or single GWAS study to combine data from different research sites and from different array/sequencing experiments. In this step of a GWAS, the data are still identifiable (Figure 1, Step 3), and locally performed imputation by data protection-friendly genotype imputation servers located in the EU[45] is not always feasible.”
We agree that the section lacked organisation and focus. We reworked this part of the article to structure it more clearly. We also further expanded on some of the legal challenges.

Reviewer 2 - comment 9
Lack of focus on family privacy: I think this statement is fairly vague : "Another data protection and security challenge is that data must be kept secure for at least the duration of the data subject's life17, if not for that of close family members."
The main issue here is which family members we should focus on? What are the ethical principles for family privacy?

Our response:
Thank you for your comment. We agree that the statement is rather vague and added further details. In principal, this relates to any blood relative as long as the genetic data can reveal information about them. However, the issue hasn’t been decided yet (see page 6). We’ve reworked the following sentence and added more information on this topic on page 6 to cover this aspect:
“Another data protection and security challenge is that personal data must be kept secure either until its deleted or for at least the duration of the data subject’s life[40], if not for that of close family members. The latter could be the case for genomic data: they differ from other personal data as they are directly linked to more than one person. No final decision on the status and rights of family members under the GDPR has been reached so far, but some scholars make strong - if controversial - cases that the need for data security doesn’t diminish with the data subject’s death as far as the data reveals information about their relatives[41,42].”

Reviewer 2 - comment 10
I think Figure 1 is fairly vague in terms of risk and accuracy levels. It also oversimplifies a lot of the details. For example, I would argue following (I can be convinced otherwise): In majority of studies, meta-analysis provides very accurate results. I base this claim on the overwhelming number of studies that compared meta analysis with centralized analysis approaches. If you also look at the most recent GWAS studies, a large portion of them are meta-analysis-based studies.

Our response:
Thank you for the comment. We agree that Figure 1 is a bit vague. However, it's meant to condense and abstract a complex topic into concrete concepts. We disagree that meta-analyses provide very accurate results. As demonstrated in (https://genomebiology.biomedcentral.com/articles/10.1186/s13059-021-02562-1), in particularly in heterogeneously distributed data, meta-analysis methods' accuracy drops drastically. Real-world data typically is heterogeneously distributed - in particular when considering international genomic backgrounds. We now cite to corresponding publication in the main manuscript.

Reviewer 3 - comment 1
Given the audience of Genome Biology, I believe this work could use more precise technical detail in the discussion of (1) privacy-enhancing technologies (Box 2), and (2) the precise legal consequences of these issues. I encourage the authors to draw upon recent legal battles, such as the 23andMe lawsuit, to inform readers concretely of potential genetic leaks and their consequences. Furthermore, the discussion of PETs in Box 2 could be more precise. For instance, it is unclear what "DP adds mathematical noise to data" means. What kind of data? Aggregated data or individual data? Who is adding the noise? Furthermore, is there any legal agreement on how to choose these noise levels?

Our response:
Thank you for the comment.
The use of privacy-enhancing technologies (PETs) is not of purely academic importance but can reduce the risk of unintentional loss of genetic data, as we were recently made aware by a publicly known hacker attack on the 23andMe database. According to 23andMe, no genetic data was stolen in this data breach, but this would be a possible scenario. By implementing PETs in the future and performing these calculations on the client side, genetic calculations of family relationships, for example, could help to ensure that genetic data no longer has to be stored on central servers which are often the target of hacker attacks. Of course, it remains to be seen whether this will meet with the interest of commercial providers. As we do not wish to comment here specifically on the press release about a possible data loss at 23andMe, we have included the following in the text on page 12: “Storing genomic data on large central servers also carries the risk of this data being stolen by hackers, because in the event of a successful attack, a large amount of genomic data from a large number of individuals falls into the hands of the attacker all at once.”
We added that researchers use DP to add mathematical noise to data, individual and/or aggregated data ones in Box 2. We’ve also included a paragraph concerning data leaks and their consequences on pages 3 to 4.

Reviewer 3 - comment 2
More discussion about genetic data privacy and GWAS outside of the EU's GDPR would be appreciated. While the GDPR is the landmark law for genetic privacy, several other countries have passed laws and it'd be good to understand how the principles introduced in Box 1 apply more broadly in other legislation.

Our response:
Thank you very much for your comment. We’ve inserted some additional information regarding privacy laws in other countries, both in the Introduction and in the section regarding cross-border transfers. There are many countries that established the GDPR or have regulations that are similar to it and in which there are parallels to the GDPR principles introduced in Box 1. We believe that further and more detailed comparisons would require their own article to be of any meaningful use.

Reviewer 3 - comment 3
This is a personal opinion, and I am happy to be convinced otherwise, but I think this article could use some more hedging. The consequences of genetic data breaches are unknown and unclear, and this should be acknowledged. Some leakage will always be likely, and it should be noted that there is significant uncertainty on the consequences of genetic privacy. Lines 217-225 are applicable to a broad range of data. Many data modalities contain personally identifiable and extractable information which could be linked to, for instance, photographs. Social media profiles, which people often publish publicly, could easily be linked to photographs or other modalities. I would like to see more discussion, or would be happy to engage in a discussion with the authors, on the uniqueness and necessity of GWAS privacy.

Our response:
Thank you for your comment. We agree and have added some information regarding the general risks and uncertainties in connection with the processing of genomic data at the end of the introductory section of the article.
It’s true that sensitive data is processed in many contexts and studies. We’ve decided to limit the scope of the article on only GWAS for mainly two reasons: Firstly, we wanted to provide concrete considerations and suggestions that consider all relevant steps of a study instead of only repeating abstract discussions about the privacy concern regarding the processing of genomic data. Secondly, we chose GWAS over other study designs as it combines potentially immense medical progress and considerable risks for the data subjects since it requires both big amounts of data and especially sensitive data (genomic data that is then processed to identify diseases).

Reviewer 3 - comment 4
The following citations are omitted but are sufficiently related:
[1] Blatt, Gusev, Polyakov, and Goldwasser, PNAS 2020.
[2] Froelicher et al, Nature Communications 2021.
[3] Johnson and Shmatikov, ACM SIGKIDD 2013.
[4] Froelicher et al, IEEE S&P 2023.

Our response:
Thank you very much for this suggestion. We agree, and now reference all four publications.

**Second round of review**

**Reviewer 1**

The authors have extensively edited and improved the manuscript. My previous comments have been addressed.

I have a few remaining comments:

1. Title: Would it be better to say legal aspects "of" privacy-enhancing technologies?

2. Page 3: "In practice, pseudonymised genomic data ... can generally only be matched to a person whose genomic data are both accessible and linked to them". I don't think this is accurate, since the person can be re-identified through his or her relatives' records in online genealogy services.

3. Page 6-7: I found the edited paragraph about Data Protection Framework between the EU and the U.S. to be valuable, but it is not clear how this legal issue relates to GWAS. Even if there are no explicit provisions about genomic data in these frameworks, I would suggest that the authors discuss the implications in the context of this article.

4. Page 7: The description about hardware-based solutions based on TEE states incorrectly that the user is required to trust the service provider. TEE removes this trust given the security of the enclave.

5. Box 2 states that PETs "enable GDPR compliant data processing". This overlooks many of the nuances that the authors explained elsewhere regarding compliance and could mislead readers into thinking that using PETs is sufficient for it.

6. Box 3: Some of the recommendations provided appear to lack the necessary depth. For example, Step 5 (association testing) says only to use a federated tool if it exists and overlooks that in practice researchers would need to choose from a variety of approaches, including meta-analysis and various PETs depending on the study context. I also found it odd that for quality control and imputation, recommendation is to perform these steps locally if possible, which could limit their effectiveness. Maybe including a comprehensive guideline isn't appropriate for this review, but more edits seem needed to make sure this Box is useful to researchers as intended.

**Reviewer 2**

I have no more comments.

**Reviewer 3**

Overall, I believe the authors did a good job at responding to my major concerns. I especially appreciate that the authors took time and space to discuss the uncertainties underlying privacy risks in GWAS analyses.

I still would like to echo a previous comment I had which was also raised by Reviewer 1 that there isn’t much clarity on how the noise parameters for differential privacy would or should be chosen for GWAS. This is especially important if the authors raise this as a potential solution, as different individuals have different thresholds for an acceptable privacy risk. The US census is already having a hard time choosing a noise parameter, for instance.

**Authors’ response to reviewers**

Reviewer 1 - comment 1
Title: Would it be better to say legal aspects "of" privacy-enhancing technologies?

Our response:
We’ve changed the title accordingly.

Reviewer 1 - comment 2
Page 3: "In practice, pseudonymised genomic data ... can generally only be matched to a person whose genomic data are both accessible and linked to them". I don't think this is accurate, since the person can be re-identified through his or her relatives' records in online genealogy services.

Our response:
Thank you very much for pointing out this imprecision. We agree and have changed the sentence to include this option. It now says (changes marked in yellow): “In practice, pseudonymised genomic data - and subsequently the study results concerning this data subject - can generally only be matched to a person whose genomic data are both accessible and linked to them, unless re-identification through relatives’ records in online genealogy services is possible - e.g. because they entered it into a database for ancestry services.”

Reviewer 1 - comment 3
Page 6-7: I found the edited paragraph about Data Protection Framework between the EU and the U.S. to be valuable, but it is not clear how this legal issue relates to GWAS. Even if there are no explicit provisions about genomic data in these frameworks, I would suggest that the authors discuss the implications in the context of this article.

Our response:
Thank you for your comment. We agree and have therefore better worked out the implications for international imputation, which is currently not yet benefiting from the desired legal certainty of the Data Protection Framework. We have added the following new sentences on page 7 (changes marked in yellow):
“For this reason, GWAS researchers who want to utilise U.S. imputation servers do not benefit from the advantages, in particular the intended legal certainty, that arise from the DPF. International imputation currently remains a data processing procedure that is legally complicated and often time-consuming. In lieu of the DPF, Article 46 GDPR mandates that appropriate safeguards must be taken and the European Commission published new standard contractual clauses in June 2021, which are mandatory for new contracts from 27.09.2021[51]. This option requires more effort and time and lacks the benefit of legal certainty as to what constitutes appropriate safeguards that the DPF offers.”

Reviewer 1 - comment 4
Page 7: The description about hardware-based solutions based on TEE states incorrectly that the user is required to trust the service provider. TEE removes this trust given the security of the enclave.

Our response:
Thank you for your comment. However, we only partially agree with this: In our opinion, TEEs cannot be understood as a homogeneous concept; rather, each TEE defines its own trustworthiness and it is up to the objective assessment of whether the claim is true. The difficulties surrounding the multifaceted understanding of TEEs have already been described in many cases, for example in: Sabt et al. Trusted Execution Environment: What It is, and What It is Not, https://ieeexplore.ieee.org/abstract/document/7345265. So even if TEEs are a good approach to ensure trustworthy data processing, trust problems can also occur with this option, which have been described in the literature, for example: https://www.ncbi.nlm.nih.gov/pmc/articles/PMC8857019/; https://www.cell.com/action/showPdf?pii=S2405-4712%2821%2900374-4. We added references to these papers into the paper in order to make sure for the reader to understand what we refer to (page 7).

Reviewer 1 - comment 5
Box 2 states that PETs "enable GDPR compliant data processing". This overlooks many of the nuances that the authors explained elsewhere regarding compliance and could mislead readers into thinking that using PETs is sufficient for it.

Our response:
Thank you very much for pointing out this imprecision. We agree and changed the statement to “enable major steps towards GDPR compliant data processing”. Box 2 is now Figure 2.

Reviewer 1 - comment 6
Box 3: Some of the recommendations provided appear to lack the necessary depth. For example, Step 5 (association testing) says only to use a federated tool if it exists and overlooks that in practice researchers would need to choose from a variety of approaches, including meta-analysis and various PETs depending on the study context. I also found it odd that for quality control and imputation, recommendation is to perform these steps locally if possible, which could limit their effectiveness. Maybe including a comprehensive guideline isn't appropriate for this review, but more edits seem needed to make sure this Box is useful to researchers as intended.

Our response:
Excellent point. We agree that it is frustrating that no recommendation applies without restriction, so that a (second-best) alternative is often necessary in order to adequately fulfil the requirements of the GDPR without completely contradicting the purpose of the research. Performing the steps locally is advisable from a legal/data protection perspective but the researcher has to ultimately decide whether that is a feasible option. Each case must be considered individually, and there is (unfortunately) no one-size-fits-all recommendation. For this reason, we believe that even a comprehensive guideline for researchers would always have this shortcoming, namely that it is ultimately up to the responsible researcher to check what their processing situation looks like and what measures they can take without their research results being affected beyond an acceptable level. However, in order to draw attention to this general limitation, we have included a disclaimer at the beginning of the recommendations.

Reviewer 3 - comment 1
I still would like to echo a previous comment I had which was also raised by Reviewer 1 that there isn't much clarity on how the noise parameters for differential privacy would or should be chosen for GWAS. This is especially important if the authors raise this as a potential solution, as different individuals have different thresholds for an acceptable privacy risk. The US census is already having a hard time choosing a noise parameter, for instance. This concern can be addressed in a short edit.

Our response:
Thank you for the comment. We agree and draw attention to this difficulty in particular. We added the following information within Box 2 (now: Figure 2): “Finding a general noise parameter for reasonable differential privacy is an open problem, as different individuals whose genome-wide information was collected for different reasons, from GWAS on cancer subtypes to GWAS studies on "harmless" phenotypes such as body size, would require different thresholds. Future research is needed that takes into account individual properties and the scientific community is trying to solve this issue.”
